# Effect of Study Duration and Outcome Measurement Frequency on Estimates of Change for Longitudinal Cohort Studies in Routinely-Collected Administrative Data

**DOI:** 10.23889/ijpds.v5i1.1150

**Published:** 2020-08-13

**Authors:** A Feely, E Wall-Wieler, LL Roos, LM Lix

**Affiliations:** 1 Department of Epidemiology and Cancer Registry, CancerCare Manitoba, ON-2114 675 McDermot Avenue, Winnipeg, Manitoba, Canada R3E 0V9; 2 Department of Community Health Sciences, University of Manitoba, S113-750 Bannatyne Avenue, Winnipeg, Manitoba, Canada R3E 0W3; 3 Department of Pediatrics, Stanford University, 291 Campus Drive, Stanford, California, USA 94305-5101; 4 Manitoba Centre for Health Policy, University of Manitoba, 408-727 McDermot Avenue, Winnipeg, Manitoba, Canada R3E 3P5

## Abstract

**Introduction:**

When designing longitudinal cohort studies, investigators must make decisions about study duration (i.e. length of follow-up) and frequency of outcome measurement. This research explores these design decisions for longitudinal cohort studies constructed using routinely-collected administrative data.

**Objectives:**

To illustrate the effects of varying study duration and frequency of outcome measurement in longitudinal cohort studies conducted using routinely-collected administrative data using a numeric example.

**Methods:**

Linked administrative data from Manitoba, Canada were used. The cohort included mothers who experienced the death of an infant between April 1, 1999 and March 31, 2012 and a matched (three:one) group of mothers who did not experience an infant death. A generalized linear model was used to test for differences between groups in the non-linear (i.e. quadratic) and linear trend over time for the number of healthcare contacts. Holding sample size constant, models were fit to the data for various combinations of study duration and measurement frequency. Regression coefficient estimates and their standard errors were compared.

**Results:**

A total of 2576 mothers were included; 644 experienced an infant death and 1932 were matches. Thirteen combinations of measurement frequency (one, two, three, four periods/year) and study duration (one, two, three, four years) were investigated. As frequency increased from one to four periods/year, the standard errors of the regression coefficients for the group difference in the non-linear trend (i.e. group-time-time interaction) decreased up to 98.9%. As duration increased from one to fours years, the standard errors decreased up to 96.9%. As frequency and duration increased, the estimated regression coefficients trended toward zero. Similar results were observed for the linear trend model.

**Conclusion:**

Longitudinal cohort studies based on administrative data offer flexibility in time-related design elements, but present potential challenges. Recommendations about how to select and report design decisions in studies should be included in reporting guidelines.

## Introduction

Routinely-collected administrative data, such as physician billing claims, hospital discharge records, and family services records, have been used to construct information-rich environments worldwide. These data, which are collected for purposes other than research (i.e. for secondary purposes), typically capture information for entire populations and can be linked at the individual-level to create longitudinal profiles for studying a wide range of health and social issues [1]. Population-based cohorts to examine the long-term effects of various events and interventions are regularly created from administrative data. Using routinely-collected administrative data for longitudinal cohort studies requires thoughtful attention to various elements of the study design.

In this study, we focus specifically on decisions about the study duration (i.e. length of follow-up) and the frequency of outcome measurement. For longitudinal cohort studies that involve primary data collection, previous studies have demonstrated how decisions about these design elements can impact statistical power and the precision of regression coefficient estimates [2–4]. Raudenbush and Liu [2] and Moerbeek [3] observed that the impact of increasing study duration (holding frequency constant) on statistical power was larger than the impact of increasing the frequency of outcome measurement (holding duration constant) by the same multiplicative factor. Increasing study duration enables the capture of potential lag-effects [5], while increasing the frequency of outcome measurement allows for detection of nonlinear trends [2,3,5].

When collecting primary data for longitudinal cohort studies, there can be trade-offs between the benefits and costs of increasing study duration and frequency of outcome measurement. Increasing the frequency of outcome measurement can be costly in terms of staff time and resources and can place a substantial burden on study participants in terms of their time commitment and potential for response fatigue [2,3,5]. A longer duration also increases study costs because participants will be followed for longer periods. There is the potential for increased participant attrition when study duration is increased; this may be due to illness, death or loss of interest in continued participation [2,3,5].

In longitudinal cohort studies that use routinely-collected administrative data there are some different considerations with respect to study duration and the frequency of outcome measurement than in studies that use primary data collection. The costs of data collection (i.e. extraction of records from a data repository) are unlikely to change as study duration increases. Participant response burden is no longer a relevant issue. However, increasing study duration may result in a decreased cohort size as individuals are lost to follow up because of death, migration, and/or loss of health insurance coverage [1]; this could also introduce selection bias into the study [6]. Decreased cohort size may negatively impact statistical power and precision of estimates of change. In some cases, such as where an increased number of outcome events results from a longer study duration, precision may improve even when cohort size decreases. In addition, a greater number of outcome measurements in a fixed time window may result in sparse event counts at each measurement occasion, which in turn can lead to challenges when modelling binary and count data.

To our knowledge, little, if any, research has described the considerations about study duration and frequency of outcome measurement for longitudinal cohort studies using routinely-collected administrative data. This paper examines the impact and potential challenges associated with these design decisions. This is accomplished with a numeric example that uses routinely-collected administrative data from Manitoba, Canada.

## Methods

The numeric example illustrates the impact of varying the study duration and frequency of outcome measurement on the ability to detect differences in change over time in the number of healthcare system contacts for two groups. Following from the literature [2–4], we expected that while holding sample size constant, increasing the study duration (holding frequency constant) and increasing the frequency of outcome measurement (holding duration constant) would decrease the standard errors of the estimated model parameters, resulting in more precise effect estimates.

Administrative data from the Population Research Data Repository housed at the Manitoba Centre for Health Policy were used for the numeric example. The Repository contains anonymized individual-level data routinely collected over time for virtually all residents of the province of Manitoba, Canada; these data can be linked using a de-identified (scrambled) unique personal health number [7]. Population registry data were linked with physician claims, hospital discharge abstracts, and pharmaceutical dispensation records. Children can be linked to their mothers using hospital abstract information captured at the time of birth [8].

The data were from a previously-published study [9]. The cohort included all Manitoba women whose first child was born between April 1, 1999 and March 31, 2012. The cohort was comprised of two groups. Group 1 included all women who experienced the death of an infant (< one year old) before March 31, 2012. The index date for Group 1 was defined as the date the infant died. Group 2 included a matched (three:one) group of mothers who did not experience the death of a child. More detailed information on the matching procedure, as well as cohort formation and exclusion criteria are provided elsewhere [9].

The outcome of interest was the total number of healthcare contacts for any reason (i.e. sum of the number of physician visits, hospitalizations, and pharmaceutical dispensations) for the mother. Given that the total number of contacts was high, the distribution was approximately normal in shape. A generalized linear model with generalized estimating equations was used to model the trend in the number of healthcare contacts over time. We initially fit the following model to the data:

$$E(Y)=\beta_{0}+\beta_{1}Group+\beta_{2}Time+\beta_{3}(Time\times Time)+\beta_{3}(Group\times Time)+\beta_{3}(Group\times Time\times Time)$$

where E(Y) denotes the expected value of the outcome and the β coefficients denote the fixed effect parameters for the main and interaction effects.

Linear and quadratic effects for time were included in the model because preliminary descriptive analyses suggested the potential presence of a non-linear trend. The three-way group-time-time interaction was included in the model to test for a difference between groups in the magnitude of the non-linear trend over time. We also fit a second, more parsimonious model to the data that included only the main effects of group and time and the two-way group-time interaction. This model was used to test the difference between groups in the linear trend over time; it was selected because it is a plausible follow-up model if there is no statistically significant evidence of a non-linear trend, which was the case for some models fit to these data. A compound symmetric covariance structure was specified to account for the correlation introduced by the repeated measurements.

Models were fit to the data for various combinations of study duration and frequency of outcome measurement. Study duration was defined as the number of follow-up years after the index date (i.e. one year, two years, three years, and four years). Frequency of outcome measurement was defined as the number of observation periods in each year of follow-up. Frequencies of one period per year, two periods per year, three periods per year, and four periods per year were included; this corresponds to observation periods of 12 months, six months, four months, and three months duration, respectively. When estimating the quadratic term for time, only combinations with at least three outcome measurement occasions were used.

Regression coefficients and their standard errors were estimated for each model. All analyses were performed using SAS version 9.4. This research was approved by the University of Manitoba Health Research Ethics Board (H2013-164) and access to the anonymized administrative data was approved by the Health Information Privacy Committee at Manitoba Health, Seniors and Active Living (HIPC# 2013/2014-04).

## Results

In total, 2576 mothers were included in the analyses; 644 of the mothers experienced the death of an infant (Group 1) and 1932 mothers who had not experienced an infant death were matched to this group (Group 2). The sample size was held constant for all combinations of study duration and outcome measurement frequency in the models that were fit to the data.

Table 1 contains the estimated regression coefficients and standard errors for each combination of study duration and outcome measurement frequency for the model that included the quadratic time effect. The highest-order interaction, the group-time-time interaction, was statistically significant for all models, with the exception of the models that were fit to three years of data and included one or two measurement periods per year. The magnitude of the estimated regression coefficients for the three-way interaction decreased as the frequency of the outcome measurements increased, holding duration constant. However, the sign of the estimated regression coefficients for the group-time-time interaction also changed. For example, with a single year of data and either three or four measurement periods per year, the estimated regression coefficients for the group-time-time interaction were positive, indicating that Group 1 had a higher estimated non-linear (i.e. quadratic) rate of increase in the mean number of healthcare contacts over time than Group 2. Additionally, the time-time interaction term was positive and statistically significant, indicating that the rate of increase was greater at periods further from the index date than those closer to the index date. However, when study duration increased to two years, the estimated regression coefficients for the group-time-time interaction were always negative, indicating that Group 1 had a lower estimated non-linear (i.e. quadratic) rate of increase than Group 2. When the study duration was three years, the estimated regression coefficients for the group-time-time interaction could be either positive or negative depending on the frequency of outcome measurements, while a study duration of four years resulted in negative estimates.

**Table 1: Estimated regression coefficients (standard errors) for a generalized linear model applied to administrative health data for combinations of study duration and frequency of outcome measurements per year; model contained coefficients to estimate the quadratic trend over time:** Note: Bold values are statistically significant at α=0.05

Duration (years)	Freq	Estimated Regression Coefficients (Standard Errors)				

		Group	Time	Time-Time	Group-Time	Group-Time-Time
1	3	2.39 (0.61)	-2.73 (0.27)	0.59 (0.07)	-0.76 (0.67)	0.35 (0.16)
	4	1.98 (0.36)	-1.67 (0.12)	0.29 (0.02)	-0.70 (0.30)	0.22 (0.06)

2	2	0.13 (0.62)	-1.28 (0.21)	0.27 (0.04)	3.77 (0.56)	-0.84 (0.11)
	3	0.98 (0.34)	-0.61 (0.08)	0.09 (0.01)	1.18 (0.22)	-0.19 (0.03)
	4	0.80 (0.24)	-0.38 (0.04)	0.04 (<0.00)	0.66 (0.11)	-0.08 (0.01)

3	1	9.76 (1.55)	0.20 (0.64)	0.10 (0.16)	-2.28 (1.66)	0.10 (0.41)
	2	3.71 (0.46)	-0.20 (0.11)	0.04 (0.02)	0.03 (0.26)	-0.06 (0.04)
	3	2.42 (0.28)	-0.12 (0.04)	0.02 (0.00)	0.03 (0.11)	-0.02 (0.01)
	4	1.80 (0.20)	-0.08 (0.02)	0.01 (0.00)	0.02 (0.06)	-0.01 (0.00)

4	1	11.04 (1.09)	1.86 (0.38)	-0.34 (0.08)	-3.90 (0.86)	0.52 (0.17)
	2	4.62 (0.41)	0.28 (0.07)	-0.03 (0.01)	-0.71 (0.16)	0.04 (0.02)
	3	2.94 (0.26)	0.10 (0.03)	-0.01 (<0.00)	-0.28 (0.07)	0.01 (<0.00)
	4	2.16 (0.19)	0.05 (0.02)	<0.00 (<0.00)	-0.15 (0.04)	<0.00 (<0.00)

A similar pattern is evident in Table 2, which contains the estimated regression coefficients and standard errors for each combination of study duration and frequency of outcome measurement for the parsimonious model with the linear time effect. The estimated regression coefficients for the two-way group-time interaction were always statistically significant, but were positive for a study duration of one year and negative for a study duration greater than one year, regardless of the frequency of outcome measurements.

**Table 2: Estimated regression coefficients (standard errors) for a generalized linear model applied to administrative data for combinations of study duration and frequency of outcome measurements per year; model contained coefficients to estimate the linear trend over time:** Note: Bold values are statistically significant at α=0.05

Duration (years)	Freq	Estimated Regression Coefficients (Standard Errors)		

		Group	Time	Group-Time
1	3	1.21 (0.30)	-0.37 (0.04)	0.66 (0.13)
	4	0.87 (0.21)	-0.23 (0.02)	0.41 (0.07)

2	2	4.31 (0.38)	0.05 (0.04)	-0.41 (0.10)
	3	2.77 (0.24)	0.01 (0.02)	-0.16 (0.04)
	4	2.05 (0.18)	<0.00 (0.01)	-0.09 (0.02)

3	1	9.44 (0.77)	0.58 (0.11)	-1.89 (0.27)
	2	4.32 (0.35)	0.12 (0.03)	-0.43 (0.06)
	3	2.82 (0.23)	0.05 (0.01)	-0.19 (0.03)
	4	2.08 (0.17)	0.03 (0.01)	-0.10 (0.02)

4	1	8.42 (0.70)	0.15 (0.08)	-1.28 (0.19)
	2	3.93 (0.33)	0.03 (0.02)	-0.30 (0.04)
	3	2.58 (0.22)	0.01 (0.01)	-0.13 (0.02)
	4	1.92 (0.16)	<0.00 (<0.00)	-0.07 (0.01)

Figure 1 displays the standard errors for the estimated regression coefficients of the group-time-time interaction in the generalized linear model that included the quadratic time effect for the various combinations of study duration and outcome measurement frequency. As the frequency increased (moving across horizontal axis), the standard error decreased. Increasing the frequency of outcome measurements from one period per year to four periods per year decreased the standard error by 98.9% and 98.7% when study duration was held constant at three years and four years, respectively. Similarly, as study duration increased (moving down vertical axis), the standard error also decreased. Increasing the study duration from one year to four years decreased the standard error of the group-time-time interaction term by 96.9% and 96.5% holding constant the frequency of outcome measurement and using three periods per year and four periods per year, respectively.

**Figure 1: Standard errors for the estimated regression coefficients of the Group-Time-Time interaction term for combinations of study duration and frequency of outcome measurement:**
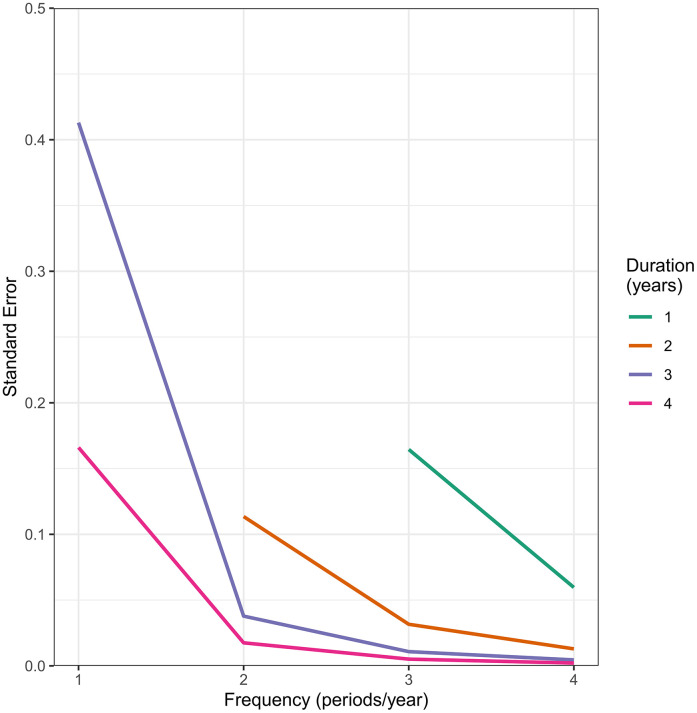


Figure 1 also reveals that the reductions in the standard errors diminished as duration and frequency increased. This suggests that at some point increasing study duration or frequency of outcome measurement may not add substantial value in terms of reducing standard errors, although these factors may still be important for capturing lag-effects and nonlinear trends and for other substantive reasons. The standard errors of the remaining terms in the model also displayed a similar pattern to that shown in Figure 1.

Figure 2 depicts the estimated regression coefficients of the group-time-time interaction in the generalized linear model that included the quadratic time effect for various combinations of study duration and frequency of outcome measurement, while holding sample size constant. As the number of time points increased by increasing study duration or frequency of outcome measurement, the estimated regression coefficient tended towards the null value. The largest change in the estimated coefficient occurred between one and two periods per year. This pattern was also evident for the remaining linear and quadratic terms in the model.

**Figure 2: Estimated coefficients of the Group-Time-Time interaction term for combinations of study duration and frequency of outcome measurement:**
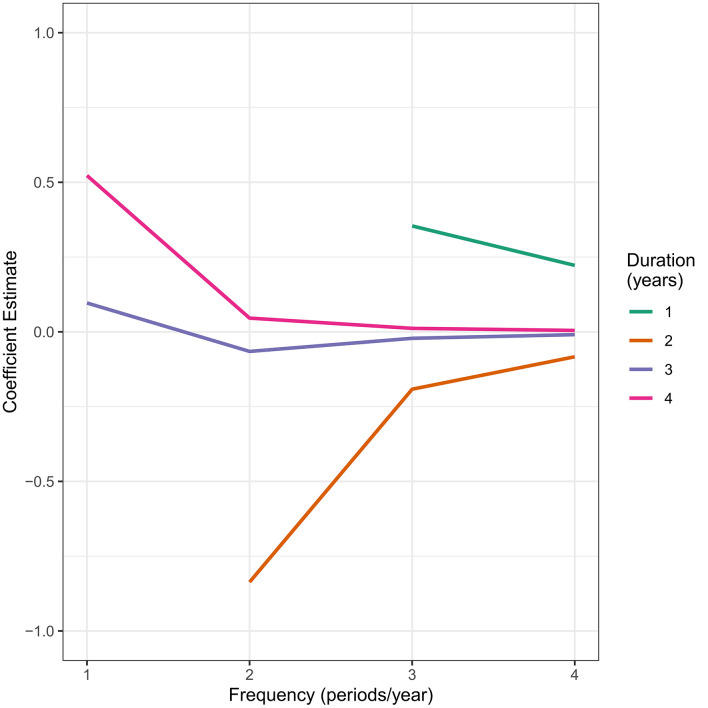


## Discussion

The numeric example illustrates the impact that varying either the study duration or the frequency of outcome measurement has on both the estimated regression coefficients and their standard errors in a longitudinal cohort study using routinely-collected, secondary administrative data. Researchers must carefully plan their study design to ensure it is best suited to their objective(s). Using routinely-collected administrative data provides flexibility in choosing and comparing duration and frequency design elements. However, researchers need to be cautious of data snooping or “cherry-picking” to find results that strongly support their research hypotheses. These practices raise ethical concerns and could produce biased results [10].

We have illustrated the effects of varying the frequency of measurement of the outcome of interest and study duration on the magnitude of time-related coefficients and their standard errors. However, we emphasize the importance of designing a longitudinal cohort study in consultation with experts in the substantive area of the research, to ensure the relevance of the study duration and frequency of measurements to the study objectives and the anticipated nature of change in the outcome of interest.

In this numerical example, we selected a marginal model to fit to the data. However, a random effects model could have also been applied to the data. The marginal model expresses population averaged relationships, whereas the random effects model expresses relationships conditional on having certain individual characteristics modelled by the random effects. Regardless of the choice of models, the same considerations about study duration and frequency of outcome measurement must be made.

Guidelines to ensure transparency in the reporting of research studies include the REporting of studies Conducted using Observational Routinely collected health Data (RECORD) statement, a checklist of reporting guidelines for observational studies using routinely-collected data, such as administrative data [11]. The RECORD statement includes many items related to study design, but does not include explicit reporting guidelines for time-related design elements in longitudinal cohort designs.

Collins and Graham [5] discuss the importance of explicitly justifying a study’s temporal design (i.e. study duration and frequency of outcome measurement) based on the features of the phenomena of interest, which should include how the design choices may affect the analytic results. However, they note that unlike other aspects of study design, researchers do not generally report the underlying rationale for their temporal design decisions.

Given the influence that variations in study duration and frequency of outcome measurement can have on the results of longitudinal cohort studies, recommendations regarding how to make and report time-related design decisions in longitudinal cohort studies using routinely-collected administrative data should be added to reporting guidelines. These decisions need to be clearly described so the research community can evaluate whether there is adequate justification for the time-related design choices that have been made.

We recognize that our study has limitations. This research relied on a single numeric example to identify the effect of time-related design decisions on study outcomes. Computer simulation could also have been used to illustrate these effects across a variety of data-analytic conditions. A matched cohort design was used in the numeric example to illustrate the effects of varying study duration and frequency of outcome measurement. This design was chosen because it was consistent with the design used in the original study for which the data were derived [9]. Depending on the research question, other observational study designs can be applied to administrative data. For example, if a rare outcome is of interest, a case-control design may be appropriate [12]. Differences in how time-related design decisions affect the results of different observational study designs could be further explored.

## Conclusion

In summary, our numeric example illustrates that decisions about study duration and frequency of outcome measurement can impact the findings of longitudinal cohort studies conducted using routinely-collected administrative data. Longitudinal cohort studies that use administrative data offer flexibility in many aspects of study design, including time-related design elements. However, time-related design decisions could present potential challenges in terms of study biases and statistical modelling. Design decisions in longitudinal cohort studies using routinely-collected administrative data should be included in reporting guidelines for research results.

## Acknowledgments

The authors acknowledge the Manitoba Centre for Health Policy for use of data contained in the Manitoba Population Research Data Repository under project #H2013-164 (2013/2014-04). The results and conclusions are those of the authors and no official endorsement by the Manitoba Centre for Health Policy, Manitoba Health, or other data providers is intended or should be inferred. Data used in this study are from the Manitoba Population Research Data Repository housed at the Manitoba Centre for Health Policy, University of Manitoba and were derived from data provided by Manitoba Health.

## Statement on Conflicts of Interest

The authors declare they have no conflict of interest.

## Ethics statement

This research was approved by the University of Manitoba Health Research Ethics Board. Access to the anonymized administrative health data was approved by the Manitoba Health Information Privacy Committee of Manitoba Health, Seniors and Active Living. Study cohort members were not required to provide consent for participation in this study, as allowed under provincial privacy legislation.
